# Safety Evaluation of Fine Bubble Shower Washing for Patients With Atopic Dermatitis: A Double‐Blind, Randomized, Prospective Crossover Clinical Trial

**DOI:** 10.1111/1346-8138.70117

**Published:** 2025-12-17

**Authors:** Hiroshi Kato, Risa Tamagawa‐Mineoka, Eiji Nakatani, Kenichi Yoshimura, Yukiko Yasui, Kasumi Kato, Motoki Nakamura, Ako Kurachi, Soshi Takeda, Akimichi Morita

**Affiliations:** ^1^ Department of Geriatric and Environmental Dermatology Nagoya City University Graduate School of Medical Sciences Nagoya Japan; ^2^ Department of Dermatology, Kyoto Prefectural University of Medicine Graduate School of Medical Science Kyoto Japan; ^3^ Department of Biostatistics and Health Data Science, Graduate School of Medical Science Nagoya City University Nagoya Japan; ^4^ MTG Co., Ltd Nagoya Japan

**Keywords:** atopic dermatitis, crossover studies, double‐blind method, fine bubble, microbubbles, randomized clinical trials

## Abstract

Atopic dermatitis (AD) involves chronic eczema resulting from barrier dysfunction. Fine bubble (FB) technology generates microbubbles (< 100 μm) and ultrafine bubbles (< 1 μm) for surfactant‐sparing cleansing. We assessed the short‐term safety of an FB shower in AD. In this double‐blind, randomized, crossover study, adults with mild AD completed two 2‐week periods separated by a 2‐week washout in sequence. Bathing instructions and petrolatum moisturizer use were standardized and enforced. The Eczema Area and Severity Index (EASI) was scored using whole‐body photographs by a blinded team. Transepidermal water loss (TEWL) and stratum corneum hydration were measured on Days 0, 14, and 28. Because baselines were unavailable for the second 2‐week period, the primary analysis compared Day 0–14 changes between groups using baseline‐adjusted analysis of covariance; Day 0–28 changes were also explored. The primary outcomes were EASI changes; TEWL and hydration were the secondary outcomes. Groups used a conventional shower (control) first and then FB shower second or vice versa (once each). Twenty‐three participants were analyzed (mean age 40.9 ± 8 years; 83% male). Day 0–14 EASI changes did not differ between FB and control (0.62 ± 2.21 vs. 0.05 ± 0.68; F = 0.93, *p* = 0.35). EASI changes to Day 28 were nonsignificant (0.02 ± 1.65 vs. −0.03 ± 1.02; *p* = 0.90). TEWL changes for Days 0–14 (0.31 ± 4.75 vs. 1.09 ± 6.21 g/m^2^/h) and to Day 28 (5.49 ± 14.43 vs. −0.27 ± 5.28 g/m^2^/h) showed no between‐group differences. Hydration changes were similar for Days 0–14 (5.53 ± 13.23 vs. 6.14 ± 7.96 AU) and to Day 28 (18.41 ± 10.33 vs. 21.09 ± 11.07 AU). No serious adverse events or discontinuations for worsening symptoms occurred. Under standardized, low‐irritant conditions, the FB shower was well‐tolerated by adults with mild AD and did not worsen severity or barrier indices over 4 weeks. However, the superiority of FB to a conventional shower was not demonstrated.

## 1 Introduction

Atopic dermatitis (AD) is a chronic inflammatory skin disease characterized by recurrent eczema and itching, in which epidermal barrier dysfunction plays a central role [1]. Cleansing is an essential component of AD management as it removes sweat, allergens, and microorganisms such as *Staphylococcus aureus* , which can exacerbate symptoms [2, 3]. However, excessive soap use, high water temperatures, and mechanical irritation may further impair the skin barrier [4, 5].

Fine bubble (FB) technology produces microscopic (< 100 μm) and ultrafine (< 1 μm) bubbles with a large surface area and negative surface charge [6]. These physical properties allow FBs to adsorb and remove oils, dirt, and microorganisms as well as penetrate pores and hair follicles without relying on surfactants. Laboratory and industrial applications have demonstrated high cleaning efficiencies and moisturizing effects. In animal studies, FB showers have reduced inflammation, increased barrier‐related proteins such as filaggrin and loricrin, and reduced the skin burden compared to conventional cleansing [7].

Despite these promising findings, clinical evidence regarding the efficacy of FB showers in patients with AD is lacking. Therefore, the aim of this study was to evaluate the safety of FB showers in patients with mild AD, with an exploratory assessment of their effects on disease severity and skin barrier function.

## 2 Methods

### 2.1 Patients

This trial included patients with AD aged 20–79 years who met the diagnostic criteria of the Japanese Dermatological Association AD treatment guidelines. Patients were excluded if they had moderate‐to‐severe AD (an Eczema Area and Severity Index [EASI] score of ≥ 7.1), were currently using medications other than moisturizers (e.g., topical steroids, immunosuppressants, or systemic therapy), were pregnant, or were of childbearing age.

### 2.2 Study Design

**FIGURE 1:**
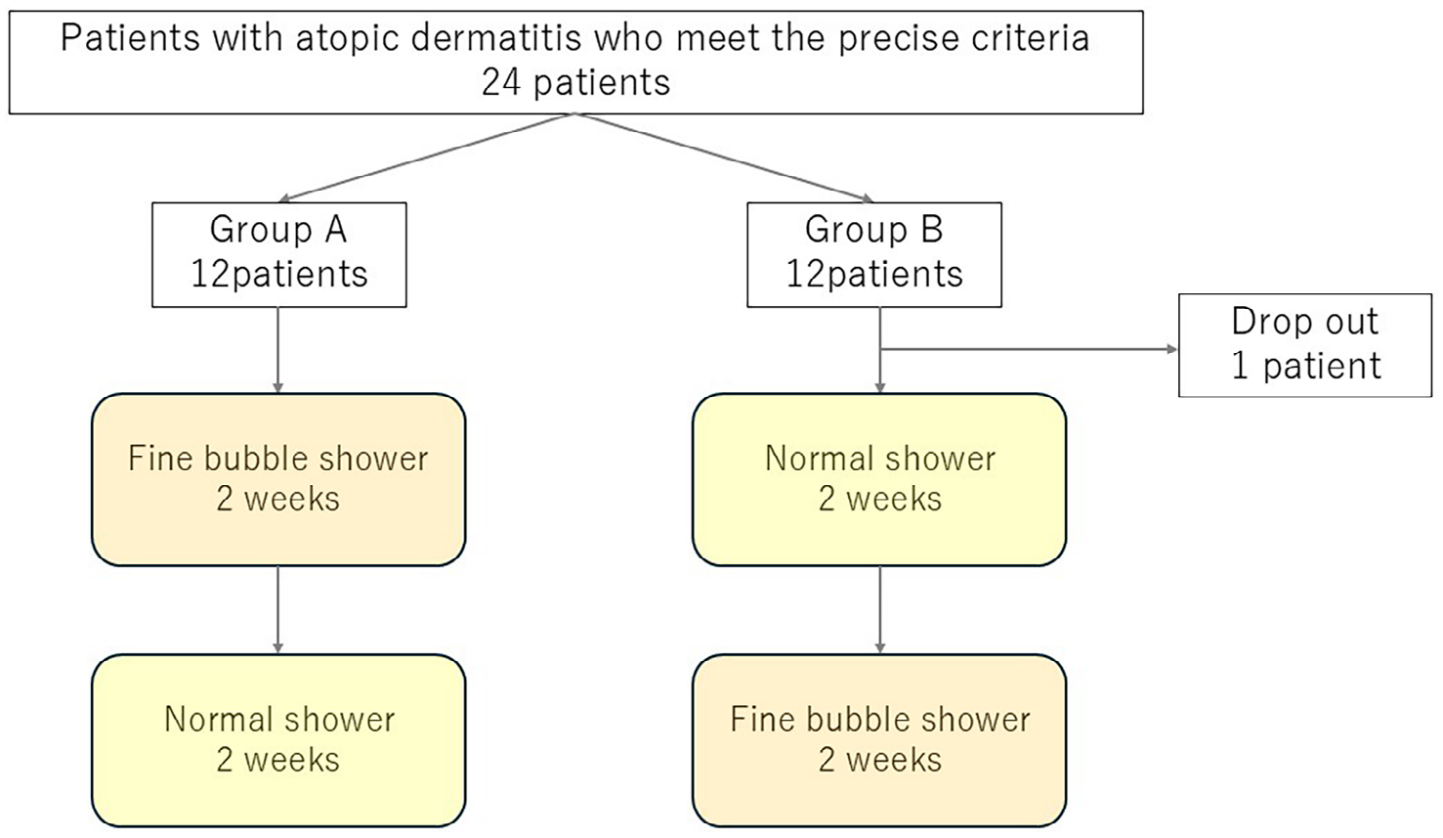
Consort flow diagram of this study. Twenty‐four participants were initially enrolled; however, one withdrew from the study and was excluded, resulting in a final evaluation of 23 participants.

This randomized, double‐blind, prospective, crossover intervention study was conducted at a single center. The participants were randomly assigned to two groups. Group A used an FB shower in the first half of the study and a conventional shower in the second half. Group B used a conventional shower in the first half and an FB shower in the second half (Figure 1). The showers looked identical; thus, participants were unable to discern the difference between the two types. Each intervention period lasted 2 weeks, and each participant underwent a total of 4 weeks of intervention (shower use), experiencing both shower conditions in a crossover design. A 2‐week washout period was established prior to intervention, during which time the participants used only petroleum jelly as a moisturizer and discontinued all other topical therapies. Baseline measurements and evaluations were conducted at the end of the washout period (Day 0). Subsequently, participants were randomly assigned to their respective groups and continued bathing under their assigned shower conditions for the first 2 weeks, starting from Day 1. An interim evaluation was conducted at the end of the 2nd week (Day 14). The participants then switched to the other shower condition and continued bathing for an additional 2 weeks. Final evaluations were conducted at the end of the 4th week (Day 28). The intervention content was designed to be accessible to participants and evaluators during the study period. Clinical evaluations (EASI scoring) were conducted by a third‐party institution (Department of Dermatology, Kyoto Prefectural University of Medicine) using photographs provided under blinded conditions. This study was conducted with the approval of the Nagoya City University Medical Research Ethics Review Committee (46‐24‐0003), and written informed consent was obtained from all participants. Ethics review management number: 46‐24‐0003. This study is registered with the Japan Registry of Clinical Trials under the registration number jRCT1042250090.

### 2.3 Trial Protocol

At the start of intervention (Day 0 following the washout period), the average baseline EASI score was 1.5 ± 1.1 in both groups. All cases were within the mild range, with no significant difference between groups. No significant differences in baseline transepidermal water loss (TEWL) or stratum corneum moisture content were observed between the two groups. None of the participants had received any AD treatment other than moisturizers for at least 2 weeks before the trial, and their condition was stable. The FB showerhead used in this study was the “ReFa FINE BUBBLE U,” manufactured by MTG Co. Ltd. (Nagoya, Japan). A showerhead of the same shape but with the FB generation function turned off was used as a control (special product, MTG Co. Ltd.). Participants were instructed to use the assigned showerhead in their home bathroom and to take a bath or shower once daily throughout the study period; bathing methods were standardized for all participants. Specific instructions included using a low‐irritation body soap (Curel Body Wash, Kao Corp., Tokyo, Japan), cleansing gently with their fingers, not rubbing with towels or sponges, maintaining a mild water temperature (approximately 38°C–40°C), showering or bathing for 5–10 min, and avoiding bath additives. After bathing, the participants were instructed to gently pat their entire body dry and apply white petroleum jelly as a moisturizer. The skincare procedures described above were consistent for all participants throughout the trial period. The only difference was the type of showerhead used (standard or fine bubbles). Note that FB showers have no special operational precautions and should be handled in the same manner as regular showers. Both groups visited the clinic once at the end of the first 2 weeks of the intervention (Day 14) for evaluation and exchange of equipment. Participants then continued bathing under opposite shower conditions for the remaining 2 weeks.

The primary outcome measure was the change in EASI score, which indicates AD clinical severity. The EASI evaluates four symptoms—erythema, infiltration/papules, lichenification/crusting, and erosion/scaling—in the head, neck, and trunk regions, as well as in the extremities, on a scale of 0 to 3. A total score ranging from 0 to 72 was calculated after adjusting for body surface area. For each participant, whole‐body photographs were taken at three time points: baseline (Day 0), intermediate (Day 14), and final (Day 28). The EASI was scored by a specialist using these photographs. However, as no pre‐intervention baseline measurement was available for the second period (Days 14–28), a standard crossover analysis was not feasible. Therefore, a reference analysis was conducted based on the change from Day 0. For the safety evaluation, we focused on confirming that no symptom worsening (EASI exacerbation) occurred during or after the trial. An increase in EASI score from the baseline to moderate or severe severity was defined as the endpoint of trial discontinuation.

Secondary outcome measures included changes in indicators of skin barrier function, specifically changes in TEWL and stratum corneum moisture content. TEWL and stratum corneum moisture content were measured using devices (Tewameter TM Hex and Corneometer CM825) manufactured by Integral Co. Ltd. (Tokyo, Japan). Measurements were taken at three specific sites on the outer side of the upper arm at each evaluation time point, resulting in a total of six points. The measurement values at each time point were averaged across the six points. All measurements were conducted in an air‐conditioned room (temperature: 20°C–25°C; humidity: 50%–70%) after 30 min of acclimatization (sitting quietly in the room). Additionally, participants were interviewed about changes in their subjective symptoms (e.g., itching or dryness) and adverse events at each time point.

### 2.4 Statistical Analysis

Because baseline measurements were unavailable during the second phase (Days 14–28), a crossover analysis could not be performed. Therefore, the study was analyzed as a quasi‐randomized controlled trial, considering the first phase (Days 0–14) as a parallel group comparison. T‐tests were used for continuous variables, and chi‐square tests were used for categorical variables to perform intergroup comparisons of patient background characteristics. The primary endpoint (change (Δ) between Day 14 and Day 0) was analyzed using analysis of covariance (ANCOVA) with baseline values as covariates to test for group differences. The target variables were the EASI score, TEWL, and stratum corneum moisture content. The same procedure was applied to all three variables. As a supplementary analysis, the cumulative change (between Day 28 and Day 0) was calculated up to the end of the second period (Day 28). All tests were two‐sided, with a significance level of *p* < 0.05. Analysis was performed using EZR, version 1.68 (Jichi Medical University, Tochigi, Japan) (EN1). As this study was not a confirmatory trial, no sample size calculation was performed, and no multiple testing adjustment was applied.

## 3 Results

### 3.1 Patient Background

Twenty‐four eligible patients were enrolled and randomly assigned to either Group A (*n* = 12) or Group B (*n* = 12). No significant differences were observed between the groups in terms of age, sex ratio, baseline EASI score (median 1.3, range 0.0–4.8), TEWL (14.0 ± 6.5 g/m^2^/h), or stratum corneum moisture content (30.9 ± 12.9 AU) (Table 1). The assignment method was as follows: case numbers were assigned in the order of patient enrollment, with odd numbers assigned to Group A and even numbers assigned to Group B. One patient dropped out during the study, resulting in 23 patients (12 in Group A and 11 in Group B) for the final analysis. The background characteristics of participants in each group are presented in Table 1. The mean age was 42.8 ± 10.8 and 38.7 ± 10.1 years in Group A and Group B, respectively, with no significant difference. The sex distribution was 67% male and 33% female in Group A, and 100% male and 0% female in Group B. Each participant was instructed to shower and bathe daily, and compliance was monitored. Monitoring confirmed that all participants complied with shower and bathing frequency requirements.

**TABLE 1:** Characteristics of participants in Groups A and B.

Variable	Category or unit	Group A	Group B	*p*
Sex	Male	8 (66.7)	11 (100.0)	0.09
	Female	4 (33.3)	0	
Age	1 year	42.8 ± 10.8	38.7 ± 10.1	0.36
EASI	1 point	1.5 ± 1.3	1.5 ± 1.0	0.96
Keratinocyte hydration	1 point	13.8 ± 7.1	14.1 ± 5.9	0.9
TEWL	1 point	81	238	0.1

*Note:* No significant differences were observed between the two groups; however, the groups showed a sex imbalance, with eight males and four females in Group A, and all participants in Group B being male.

Abbreviations: EASI, Eczema Area and Severity Index; TEWL, transepidermal water loss.

### 3.2 Changes in EASI Scores

**FIGURE 2:**
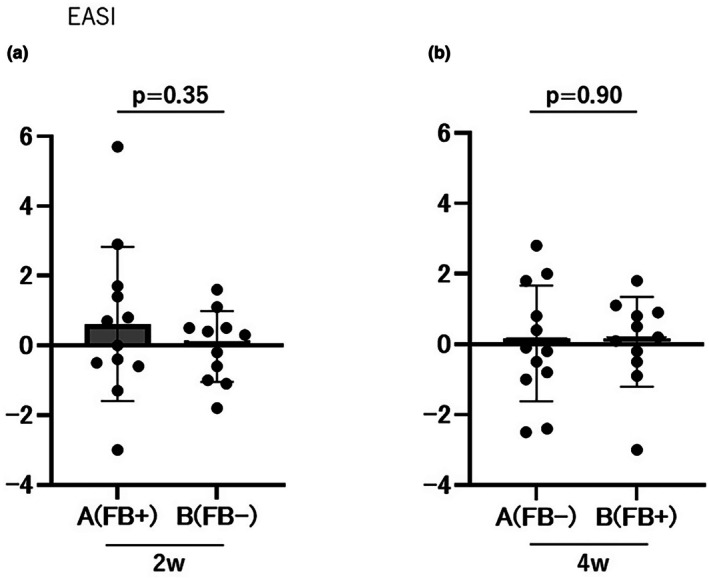
Evaluation of EASI scores. No significant differences were observed between Groups A and B in the 2‐week (a) or 4‐week (b) evaluations. EASI, Eczema Area and Severity Index.

In phase 1, the change in EASI scores (Day 14 minus Day 0) was 0.62 ± 2.21 for Group A and 0.05 ± 0.68 for Group B, with no significant difference between the groups, as determined by ANCOVA (*F* = 0.93, *p* = 0.35; Figure 2a). As a supplementary analysis, the cumulative EASI change (Day 28 minus Day 0) was 0.02 ± 1.65 in Group A and −0.03 ± 1.02 in Group B, with no significant difference between groups (*p* = 0.90; Figure 2b).

### 3.3 Changes in Skin Barrier Function Indices

**FIGURE 3:**
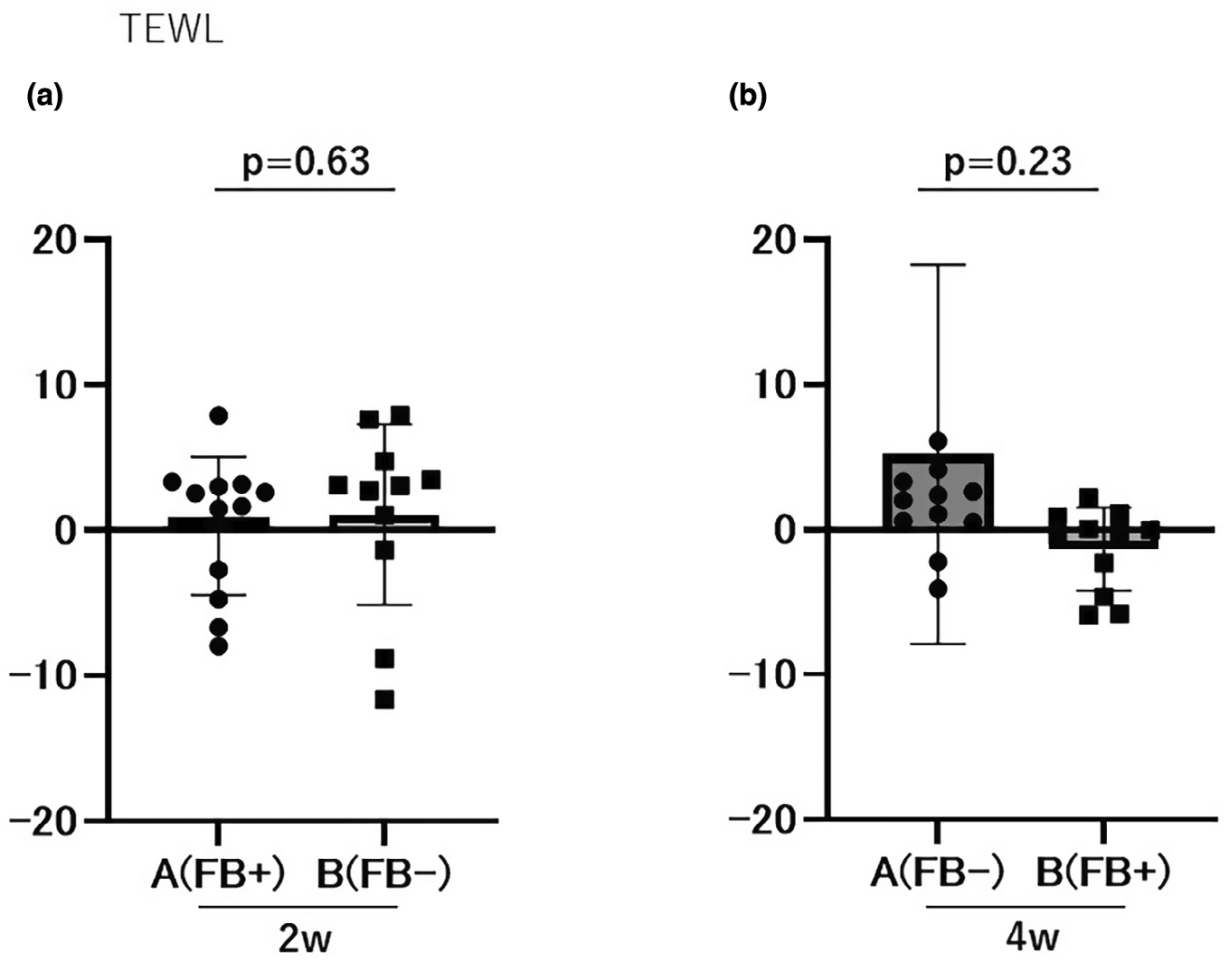
Evaluation of TEWL scores. No significant differences were observed between Groups A and B in the 2‐week (a) or 4‐week (b) evaluations. TEWL, TransEpidermal Water Loss.

During the first phase, TEWL changes were 0.31 ± 4.75 g/m^2^/h in Group A and 1.09 ± 6.21 g/m^2^/h in Group B (*p* = 0.63; Figure 3a). The cumulative TEWL change was 5.49 ± 14.43 g/m^2^/h in Group A and −0.27 ± 5.28 g/m^2^/h in Group B (*p* = 0.23; Figure 3b). No significant differences were observed between the groups.

**FIGURE 4:**
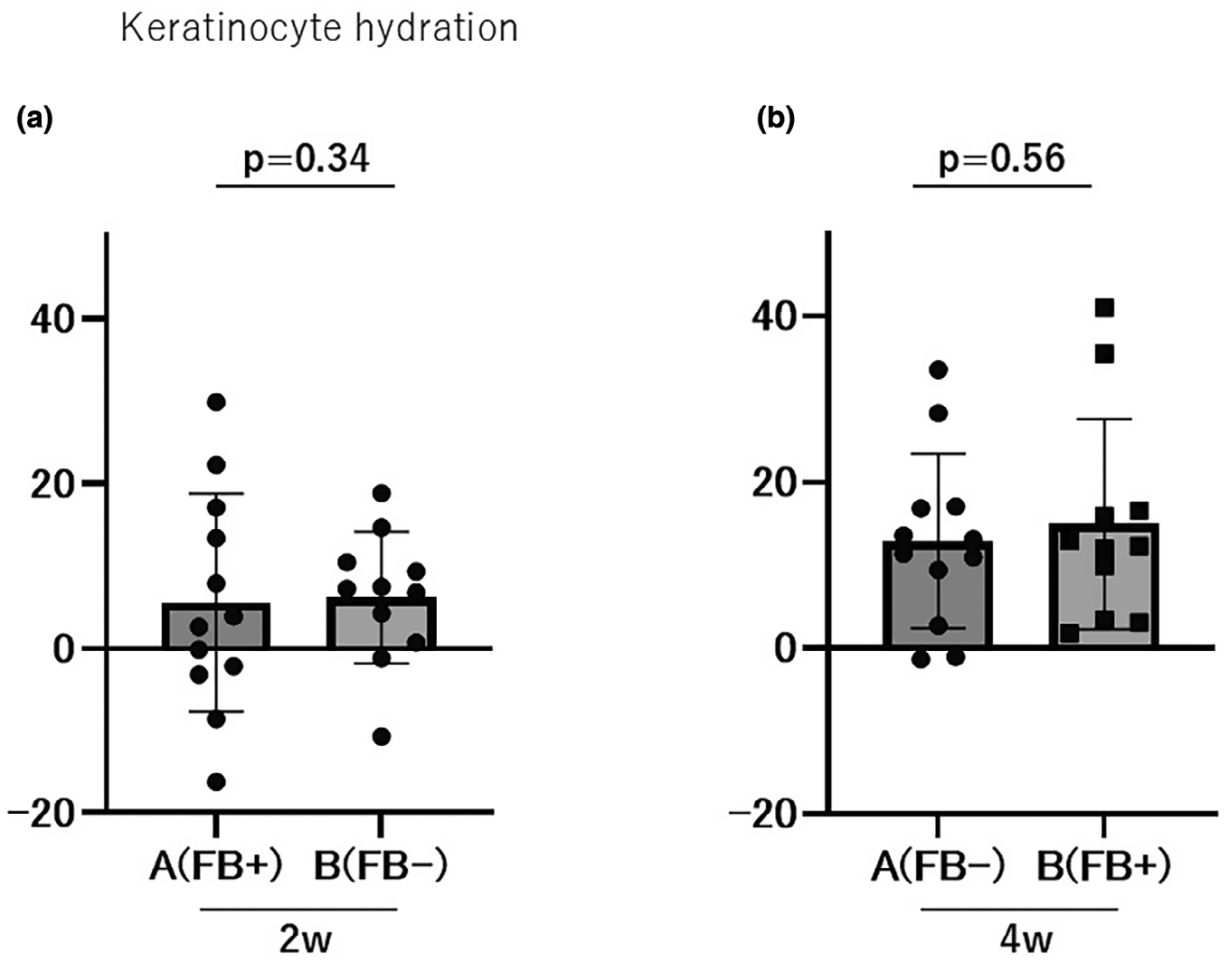
Evaluation of keratinocyte hydration scores. No significant differences were observed between Groups A and B in the 2‐week (a) or 4‐week (b) evaluations.

The change in stratum corneum moisture content was 5.53 ± 13.23 AU in Group A and 6.14 ± 7.96 AU in Group B during the first period (Days 0–14; *p* = 0.34; Figure 4a). The cumulative changes in stratum corneum moisture content were 18.41 ± 10.33 AU and 21.09 ± 11.07 AU in Group A and Group B, respectively (*p* = 0.56; Figure 4b), with no significant difference between the groups.

### 3.4 Adverse Events

No serious adverse events occurred during the study period and no participants discontinued the study owing to specific skin abnormalities or worsening of EASI after FB shower use.

## 4 Discussion

This study represents the first clinical validation of the safety and efficacy of FB showers as part of a daily skincare routine for patients with AD. The results demonstrated that cleansing with FB showers is safe for patients with AD and does not worsen skin lesions or disrupt skin barrier function with short‐term use. However, no significant differences were observed in the improvement of clinical symptoms (EASI) or objective skin conditions (TEWL and moisture content) compared with conventional low‐irritation shower cleansing, indicating no significant difference in efficacy between the two methods. Under the conditions of this study, the additional benefits of FB cleansing could not be clearly demonstrated.

Regarding safety, the study involved a crossover intervention for approximately 1 month in patients with mild AD, and no worsening of symptoms or severe adverse events were observed during FB shower use. This finding suggests that FB‐technology‐based cleansing is generally tolerable in patients with AD. Previous research has suggested that FB baths impose a lower skin burden than conventional shampoo therapy in a canine AD model, with weekly FB bathing for 4 weeks resulting in no harmful changes in skin TEWL or moisture content [8]. Our study confirmed these findings in human patients with AD, demonstrating that FB cleansing is at least as safe as conventional water‐based cleansing and may represent a gentler alternative to shampooing. The ability to incorporate this cleansing method into daily life without stress is important for treatment adherence and improving quality of life. Therefore, FB showers should be further evaluated as safe and well‐tolerated skincare options.

Although this study did not demonstrate the superiority of FB showers in terms of their efficacy (clinical effects), several points should be considered. First, the conventional shower bath used as a control may represent an effective intervention for AD management. As reported by Mochizuki et al., shower bathing improves skin symptoms in patients with AD [9]. The average improvement in EASI scores shown by both groups during the trial period suggests that natural remission or alleviation of AD symptoms occurred through continued appropriate cleansing and moisturization under both shower conditions. All participants in this trial strictly adhered to a standard skincare regimen involving low‐irritant cleansing and adequate moisturization; this regimen may have represented a higher quality than that previously received by the participants. Therefore, both groups benefited from enhanced basic therapy, which may have masked the unique effects of the FB. Accordingly, to detect any additional effects of FB, it may be necessary to intentionally provide inadequate care to the control group or use more sensitive outcome measures. However, from an ethical and practical standpoint, inadequate care is inappropriate; therefore, realistic control conditions (appropriate standard care) were adopted in this study. The absence of differences under these conditions can also be considered evidence that the effect of FB is not significantly superior to that of standard care.

Second, limiting the study population to patients with mild AD may have contributed to the lack of statistically significant differences. In patients with mild AD, symptom scores and barrier indicators are already close to the normal ranges, leaving little room for improvement. The average baseline EASI score was low (1.5), and many patients were already close to being in remission. Therefore, the impact of each shower may have been small and statistically undetectable. Future studies targeting patients with moderate or severe AD or those experiencing exacerbations may reveal significant differences in symptoms or barrier function indicators. However, severely ill patients may be at risk of exacerbation from the cleansing stimulus itself; therefore, careful evaluation of the risk versus benefit is necessary.

Third, the short intervention period may have hindered the detection of effects. In this study, the usage period for each shower condition was 2 weeks, which may have been too short to evaluate changes in AD skin condition or the microbiome. Previous research has suggested that changes in skin microbiota composition or chronic barrier repair effects may only be detected with several months of observation [10]. Experimental results have shown that ultrafine bubble showers reduce IL‐4/IL‐13 and increase filaggrin in an exogenous AD mouse model [7]. However, to confirm similar increases in barrier proteins in human skin, long‐term interventions or biopsy‐level evaluations are necessary. Therefore, to comprehensively evaluate the potential efficacy of FB showers, long‐term trials with extended trial periods should be combined with measurements of molecular markers of barrier function (e.g., filaggrin expression levels or ceramide levels) and skin microbiome analysis. Finally, we acknowledge a limitation of this study regarding the existence of sex differences across groups, which may have induced bias.

In summary, FB shower therapy is a safe and practical skincare method for patients with AD, although no significant difference in short‐term symptom improvement was observed compared with conventional shower therapy. These findings reaffirm the importance of skincare in the treatment of AD, whereby mild AD can be adequately controlled with basic management such as keeping the skin clean and moisturized. Adding the latest technology does not necessarily lead to immediate visible improvements; however, if safety is ensured, and patient comfort and convenience are improved, the introduction of new technologies may be meaningful. FB showers are available for general household use, making them an easily adopted self‐care method for patients. To our knowledge, this study provides the first clinical data supporting the safety of FB technology and suggests that patients with AD can use FB products with confidence. Larger‐scale trials with equal numbers of male and female participants and more evaluation outcomes should be adopted to clarify the efficacy of FB technology and its optimal application conditions, as well as determine its potential value for patients with exogenous AD and effectiveness without the use of soap. Such research could position FB technology as a new option for the daily management of AD that improves patient quality of life through non‐pharmacological therapies.

## Funding

This research was conducted with funding from MTG Co., Ltd. (joint research), and JUTAK20684 is indeed the funding number paid to Nagoya City University.

## Ethics Statement

Approval of the Research Protocol by an Institutional Review Board: This study was conducted with the approval of the Nagoya City University Medical Research Ethics Review Committee (Ethics review management number: 46‐24‐0003). Registry and the Registration No. of the Study/Trial: This study is registered with the Japan Registry of Clinical Trials (registration number jRCT1042250090).

## Consent

Written informed consent was obtained from all participants.

## Conflicts of Interest

Hiroshi Kato, Risa Tamagawa‐Mineoka, Eiji Nakatani, and Kenichi Yoshimura received research funding from MTG Co. Ltd. Hiroshi Kato received technical consulting fees from MTG Co. Ltd. Ako Kurachi and Soshi Takeda are employees of MTG Co. Ltd. MTG Co. Ltd. also provided the test equipment (a fine bubble shower head) and body soap. Care was taken to avoid bias when interpreting the content and results of the study. Hiroshi Kato serves as an advertising ambassador for Product Curel (Kao Corp.) and receives financial compensation.

## Data Availability

All authors have full access to all data in this study.
